# High-Throughput Sequencing Analysis of the Changes in the Salivary Microbiota of Hungarian Young and Adult Subpopulation by an Anthocyanin Chewing Gum and Toothbrush Change

**DOI:** 10.3390/dj11020044

**Published:** 2023-02-08

**Authors:** Boglárka Skopkó, Melinda Paholcsek, Anna Szilágyi-Rácz, Péter Fauszt, Péter Dávid, László Stündl, Judit Váradi, Renátó Kovács, Kinga Bágyi, Judit Remenyik

**Affiliations:** 1 Department of Dentoalveolar Surgery, Faculty of Dentistry, University of Debrecen, 4032 Debrecen, Hungary; 2 Institute of Food Technology, Faculty of Agricultural and Food Sciences and Environmental Management, University of Debrecen, 4032 Debrecen, Hungary; 3 Department of Pharmaceutical Technology, Faculty of Pharmacy, University of Debrecen, 4032 Debrecen, Hungary; 4 Department of Medical Microbiology, Faculty of Medicine, University of Debrecen, 4032 Debrecen, Hungary; 5 Department of Operative Dentistry and Endodontics, Faculty of Dentistry, University of Debrecen, 4032 Debrecen, Hungary

**Keywords:** sour cherry, gum chewing, toothbrush change, saliva, oral microbiota, recolonization

## Abstract

The sour cherry contains anthocyanins, which have bactericide action against some oral bacteria (*Klebsiella pneumoniae*, *Pseudomonas aeruginosa*). Sour cherry also has antibiofilm action against *Streptococcus mutans*, *Candida albicans,* and *Fusobacterium nucleatum*. Our earlier research proved that chewing sour cherry anthocyanin gum significantly reduces the amount of human salivary alpha-amylase and *Streptococcus mutans* levels. The microbiota of a toothbrush affects oral health and regular toothbrush change is recommended. A total of 20 healthy participants were selected for the study. We analysed saliva samples with 16S rRNA sequencing to investigate the effect of 2 weeks (daily three times, after main meals) of chewing sour cherry anthocyanin gum—supplemented by toothbrush change in half of our case–control study cohort—after scaling on human oral microbiota. A more stable and diverse microbiome could be observed after scaling by the anthocyanin gum. Significant differences between groups (NBR: not toothbrush changing; BR: toothbrush changing) were evaluated by log_2_ proportion analysis of the most abundant family and genera. The analysis showed that lower level of some Gram-negative anaerobic (*Prevotella melaninogenica*, *Porphyromonas pasteri*, *Fusobacterium nucleatum subsp. vincentii*) and Gram-positive (*Rothia mucilaginosa*) bacteria could be observed in the case group (BR), accompanied by build-up of health-associated Streptococcal network connections.

## 1. Introduction

Caries is a complex process affecting approximately 80% of humans living in the world. Previously the Streptococci and Lactobacilli were in the center of caries research as main causative factors. Caries and periodontal diseases can both originate from the fermentable dietary carbohydrates, as frequent consumption of acidic foods and beverages can lead to a dysbiotic oral microbiota rich in acidogenic and aciduric species [1,2]. Numerous chronic diseases are connected to the oral microbiota (e.g., inflammation related diabetes, cardiovascular, autoimmune, and inflammatory bowel disease), which confirm the role of investigations on oral microbiota [3].

According to the last Hungarian epidemiological populational research, the general DMF-T (number of decayed, missing, filled teeth of a person) status in 2008 of Hungarian young adults (age between 20–34) was 12.76 ± 5.45, and in adults (age between 35–44) it was 15.40 ± 5.13. The DMF-T status of the adult population showed a positive tendency regarding caries incidence [4]. In Hungary, the salivary microbiota was investigated in some studies regarding gingivitis and periodontitis—which compared them to healthy controls [5,6]—but it is also important to obtain metagenomic data about oral microbiota in relation to caries. 

It is known that oral microbiota has geographical characteristics which are influenced by internal and external factors (genetical, environmental, nutritional factors, stress, or the composition of tap water), but it also shows circadian oscillation [7,8]. It is also known that the healthy salivary microbiota is diverse and stable. It was proven that the core microbiota can be stable for as long as 7 years; hence, a deeper understanding of healthy core-oral microbiota is important to facilitate further biomarker studies aiding prevention and early diagnosis of caries, the bacterial taxonomical composition of the healthy oral state, as well as for better understanding the dysbiotic nature of oral diseases (caries, periodontitis) [9].

A description of the healthy core-oral condition is essential to gain knowledge about the bacterial taxonomical composition of the healthy oral state. Oral microorganisms build complex communities by sharing a common ecological niche. These organisms maintain a delicate and mutually beneficial balance. Earlier investigations found that the modules of bacterial networks in the oral cavity represent sub-communities of distinct ecological environments. The oral microbiota is responsive to biotic and abiotic changes; in that respect, a better understanding of the most important drivers of the composition of the core microbiota is needed [10].

By defining the health- and disease-associated bacteria, we can understand the background of the disease process, which can help to research and develop preventive agents. The sequencing methods target either the different regions of the bacterial ribosomal RNA (ribonucleic acid) gene [11] or their whole genomes (microbial metagenomes) [12]. The sequencing of Svedberg ribosomal ribonucleic acid, also called 16S rRNA, has more parameters for the evaluation of microbial changes in the course of treatments. One of the major parameters is the Chao-1 diversity, which is an estimator based on abundance, and means the richness of species in a sample [13,14]. Another important parameter, Weighted Unifrac analysis, shows the relative frequency of different taxa. It means the total amount of several branches found on the phylogenetic tree [15].

The fermentable/non-fermentable dietary carbohydrates in a susceptible person can disrupt the microbial balance of the oral cavity [2]. In addition to proper oral hygiene, the regular usage of chewing gums can reduce the fluctuation of the pH-value after meals with acidic attacks, and prevent the emergence of a dysbiotic oral microbiota. Homoki, et al. [16] studied the effects of a chewing gum containing anthocyanins of the sour cherry extract or placebo on human saliva, and they found that chewing the gum with sour cherry slowed the starch breakdown by the inhibition of HSA (human salivary amylase) and arrested the growth of S. mutans in the mouth. Hevesi, et al. [17] also showed that sour cherry can inhibit the growth of antibiotic resistant forms of Pseudomonas aeruginosa and Klebsiella pneumoniae.

Zinn, et al. [18] studied the toothbrush-associated microbiota, and they showed that the biggest changes in the microbial composition occur during the first two weeks after the toothbrush change, from which can be drawn a parallel with the plaque build-up. 

Our first aim was to investigate the effect of sour cherry anthocyanins on the salivary microbiota in a northeast Hungarian young, adult population. Therefore, we aimed to perform a complete 16S based phylotyping of saliva samples of our study population. 

Our second aim was to examine the effect of toothbrush change on the composition of microbiota. By this, we wanted to identify clusters in the oral microbiota having a crucial role in community resilience. Within this study, we also described the construction of the oral-core microbiota of our study population with low caries frequency.

## 2. Materials and Methods

### 2.1. Study Participants

Volunteers were selected from the University of Debrecen workers to participate in the investigation. The self-controlled, open-label study was approved by the ETT TUKEB, Hungary (licence number: IV/1120-1/2020/EKU) on human investigation. The study was conducted at the University of Debrecen’s Faculty of Dentistry in the Department of Periodontology and the Faculty of Agricultural and Food Sciences in the Institute of Food Technology, and the anthocyanin gum was prepared in the Department of Pharmaceutical Technology, in accordance with the World Medical Association Declaration of Helsinki (University Laboratory Accreditation Number: DIN EN ISO: 9001-2015 REG. NO. MQ 20-063 H/2). The clinical study is registered in https://clinicaltrials.gov/ (accessed on 6 March 2022) (protocol number: 2022-IV/1120-1/2020, ID: NCT5406011) and fulfilled the criteria of CONSORT guidelines.

Inclusion criteria: Age more than 18 years, signed consent statement, and good oral hygiene (at least 20 caries-free, natural teeth which may have restoration on only one surface). Exclusion criteria were the following: smoking, antibiotic treatment, or infective disease during the past two months; diagnosed hyposalivation; oral infection (with visible signs); serious systemic disease; mental problems; periodontal disease; pregnancy; taking oral contraceptives; allergy to lactose [19].

The patients agreed with the research circumstances that allowed them to stop participating in the experiment if they wanted to, then they gave their written consent to their participation. They were asked to inform us if they had been prescribed antibiotics because of any infection during the study period. All of the patients and their documentations were marked with numbers during the experiment to maintain the privacy of the data. The investigational sheet contains the patient’s number, initial dental status with the DMF-T index (DMF-T: number of decayed, missing, filled teeth), and initial basic periodontal examination (BPE). [20]

### 2.2. Clinical Procedures

**Figure 1:**
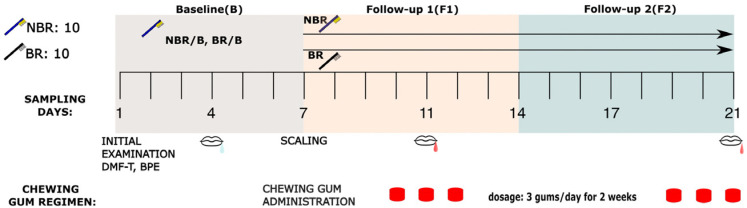
The 21 days of experimental protocol: The timescale (Periods of 1-week Baseline: B, 2-week chewing of anthocyanin gum in three pieces/day dosage. Follow-up 1 and 2: F1 and F2) of the experiment demonstrating the clinical procedures and sampling appointments. Scaling was made on the last occasion of B period, whereafter the participants started to chew the gum. After scaling made on the 7th day of B period, 10 subjects were randomly classified into NBR (who changed the toothbrush after scaling) and 10 into BR groups (subjects who did not change their toothbrush after scaling), and they got sour cherry chewing gums in three pieces/day dosage. The groups differed only by changing the toothbrush or not after scaling. The text under sampling days shows the actual intervention made at that time. During the B period (B: Day 4), sampling was made without sour cherry chewing gum usage (mouth with colorless drop). The chewing gum usage and sampling of the F1 and F2 period (F1: Day 4 and F2: Day 7) is shown by a mouth with red drop.

The study design is presented on Figure 1. It was a self-controlled experiment taking 21 days for a patient, and according to its objective, the study had consecutive parts [baseline (B), follow-up 1 (F1) and follow-up 2 (F2) periods] with defined sampling days for 16S rRNA sequencing of saliva (B: Day 4 of first week, F1: Day 4 of second week and F2: Day 7 of third week). On the sampling days the patients could eat breakfast in the morning, then wash their teeth and keep their customary dental healthcare habits. An hour before the sampling they were not allowed to eat, drink, or chew a tablet [21,22]. We collected 1-1 mL saliva from every patient during a visit into DNase free and aseptic disposable tubes. The sampling was always made between 11:00 and 14:00 to avoid diurnal variation [23]. All of the instructions were listed in the patients’ leaflet forms. 

The first week was the baseline period (B). During the first week, they were asked to maintain their routine oral hygiene habits, without any changes. In all groups after the baseline period, a professional scaling and polishing was made for every participant [it was the starting point of the follow-up 1 period (F1)]. The patients were then divided into groups based on the following aspects: Ten participants were randomly selected into a group where they did not change their toothbrush after scaling (NBR). The other half of the participants were asked to change their toothbrush (BR) to a new one after the scaling. After the scaling they were given chewing gum tablets containing sour cherry extract and asked to chew a tablet 1–5 min after tooth-brushing following the main meals. On the third week, the study continued without scaling to analyse the continuous actions of the chewing tablet with sour cherry extract [follow-up 2 (F2) period].

The participants were also divided by their age into young (aged between 18 and 30) and young adult (aged between 30 and 45) groups.

### 2.3. Statistical Analysis of DMF-T Values

Differences between the groups by toothbrush change and groups by age were checked with GraphPad Prism 9. The differences were considered significant if the *p* value was less than 0.05.

### 2.4. Anthocyanin Content and Formulation of the Chewing Gum

Formulation of the chewing gums was made by the Department of Pharmaceutical Technology. The Department has a licence to produce nutritional supplements. The sour cherry extract was prepared from the Hungarian sour cherry (*Prunus cerasus* L.) variety “VN1”, a selection of “Csengődi csokros”. The anthocyanin content was determined by Homoki and Nemes [24], where it contained mainly cyanidin 3-rutinoside, cyanidin 3-O-glucoside, delphinidin, malvidin, peonidin, and petunidin glycosides. The extract contained xylitol, chewing gum base, xylitol syrup, glycerine, citric acid, peppermint aroma, and sour cherry extract. 

The anthocyanin-containing chewing gum was made as was written in our earlier study [16]. The main ingredients were Geminis T BHA gum (Cafosa) base, xylitol, citric acid, glycerol, saccharine (Sigma), peppermint volatile oil, and sour cherry extract. During the preparation, the flavourers (citric acid, glycerol, saccharine) were placed into purified water with 0.1 g anthocyanin-containing sour cherry extract, then at 60 °C, the water phase and melted gum base were mixed, and the peppermint volatile oil was added at 40 °C. Next, 2.5 g chewing tablets were formed from the mixture, and after 12 h of conditioning at room temperature, the tablets were put into plastic boxes and stored at 8–15 °C until consumption. 

### 2.5. Saliva Sample Processing for 16S rRNA Analysis

Sample preparation. Samples according to the different parts of the experiment and different aims in each group were pooled together to obtain six libraries: NBR/B (NBR baseline), BR/B (BR baseline) [B contained altogether the NBR/B and BR/B], F1 of NBR and/or BR [follow-up 1: Day 4], F2 of NBR and/or BR [follow-up 2: Day 7]. The different pools could be grouped together by the time (B, F1, and F2) or by the two groups [NBR/B, BR/B, NBR (F1 and F2 period of NBR) and BR (F1 and F2 period of BR)]. The samples were also sequenced by the two age groups (Young and/or Young Adult: B, F1, and F2), which generated another six libraries.

The sample preparation for sequencing and sequencing data analysis were similar, as can be found in publication of Fidler, et al. [25].

### 2.6. DNA (Deoxyribonucleic Acid) Isolation and Cell Lysis 

The mechanical lysis of 250 μL saliva sample with 750 μL PowerBead Solution (Qiagen, Hilden, Germany) and 60 μL C1 Solution were vortexed and incubation was performed in the supplied Bead Tubes (Qiagen, Hilden, Germany) according to the manufacturer’s protocol in MagNa Lyser Instrument (Roche Applied Sciences, Penzberg, Germany). The vortexing and incubation were repeated once again.

DNA isolation: After lysis, DNA Isolation was performed with the commercial QIAamp PowerFecal DNA Kit (Qiagen, Hilden, Germany) according to the manufacturer’s protocol from inhibitor removing steps. 

DNA quality check: DNA concentrations were determined fluorometrically using Qubit Fluorometric Quantitation dsDNA assay kit (Thermo Fisher Scientific, MD, USA) on a CLARIOStar microplate reader (BMG Labtech, Ortenberg, Germany). All samples were diluted to 1 ng/μL with PCR (polymerase chain reaction)-grade water. Purity was assessed by measuring the absorbance at 260 and 280 nm wavelengths using Nanodrop 2000 Spectrophotometer (Thermo Fisher Scientific, MD, USA). The optimal values of the absorbance ratios were expected to be in the range of 1.7–2.0 (A260/A280) and 1.8–2.2 (A260/A230). The purified DNA samples were stored at −20 °C.

Negative and positive controls: Sterile surgical gloves and face masks (for collecting samples) were used and all DNA extraction steps were performed with sterile or sterilized equipment in a class II laminar air-flow cabinet. Negative isolation control (NIC) experiments were simultaneously conducted by substituting samples with PCR (polymerase chain reaction)-grade water. Eluted NIC samples were used for V3–V4 PCR, and indexing was performed under DNA-free ultraviolet (UV)-sterilized AirClean PCR workstations/cabinets. At each PCR clean-up step of the library preparation, NIC amplicons were also validated on a 4200 Tape Station system (G2991AA; Agilent Technologies, Santa Clara, CA, USA) using Agilent D1000 ScreenTape (5067–5365) (Santa Clara, CA, USA). For measuring the overall quality of Illumina MiSeq paired-end (PE, 2 × 301 nt) sequencing runs, 5% PhiX spike-in quality control (PhiX Control Kit v3—FC-110-3001) was used (Illumina Inc., San Diego, CA, USA).

### 2.7. Library Preparation

Standard library preparation was performed according to Illumina 16S Metagenomic Sequencing Library Preparation protocol (15044223 Rev. B). The V3 and V4 hypervariable regions of bacterial 16S rRNA gene were sequenced with Illumina MiSeq benchtop sequencer, generating ~460 bp amplicons by using the universal primer set: 341F-5′ CCTACGGGNGGCWGCAG 3′ and 785R-5′ GACTACHVGGGTATCTAATCC 3′ primers flanked by Illumina overhang adapter sequences (forward overhang: 5′ TCGTCGGCAGCGTCAGATGTGTATAAGAGACAG 3′, reverse overhang: 5′ GTCTCGTGGGCTCGGAGATGTGTATAAGAGACAG 3′) (Sigma Aldrich, St. Louis, MO, USA). After completion of the amplicon PCR with 2 × KAPA HiFi, HotStart ReadyMix dual indexing of the 96 samples (i7-N7xx-12 items, i5-S5xx-8 items) was performed using the Illumina Nextera XT Index Kit (FC-131-1001/2). PCR clean-ups and amplicon size selections were carried out with MagSI Pure Beads (KAPA Biosystems, USA, Wilmington, Massachusetts) based on the technical data sheet (KR1245—v3.16) of the manufacturer resulting in final ~550–630 bp libraries. Every time, verifications were done with PCR Agilent D1000 screen tapes (5067–5582) and D1000 Reagents (5067–5583). The 16S amplicon libraries for each sample were quantified with qPCR, normalized with respect to amplicon sizes, and pooled into a single library in equal molar quantities. Finally, 5 μL of pooled 4 nM DNA library pool was denatured with 0.2 M NaOH and diluted to 8 pM final concentration. Sequencing was carried out with MiSeq Reagent Kit v3–618 cycle (MS-102–3003) following the manufacturer’s protocols (Illumina Inc., San Diego, CA, USA) on Illumina MiSeq platform.

### 2.8. Bioinformatic Analysis

Phylogenetic analysis. After downloading raw FASTQ files QIIME 2 (version: 2021.8), pipeline (https://qiime2.org/, accessed on 1 August 2022) was used for data analysis. First, FASTQ files were imported into QIIME2 format. To remove the remainder of adapter sequences, CTGTCTCTTATACACATCT was checked and trimmed from the 3′ end of the reads with Cutadapt software (in the QIIME 2 pipeline). For quality trimming, the DADA2 software was used with the following settings: from both the forward and reverse reads, nothing was removed from the start. In the case of forward, the length was 300 bases, whilst for the reverse reads, the length was set to 256 bases [25,26].

Taxonomic alignment. Taxonomic classification of the reads was performed with Naïve Bayesian machine learning-based classifier, by using the Human Oral Microbiome Database (HOMD, http://homd.org/, ver.: 15.22, accessed on 1 August 2022). The phylogenetic trees were calculated with the align-to-tree-mafft-FastTree plugin integrated in QIIME2 pipeline [25].

Statistical analysis. QIIME 2 pipeline was used for the beta diversity analyses, and the read depth was set to 8264 to normalize the samples. To analyse beta diversity, weighted UNIFRAC distances were calculated. Beta diversity matrices were visualized with emperor plugin. Uni-frac analysis helped us in the explorative data interpretation [15]. Statistical analysis of the beta diversity was performed with pairwise PERMANOVA test. To analyse ‘alpha diversity’, Chao1 index was counted in the QIIME 2 pipeline. The significant differences were calculated with Kruskal–Wallis pairwise test [25]. 

Data visualization. Metacoder an R package was used for calculating and visualizing heat trees showing differences between different treatments or subgroups with their relative frequencies or log_2_ median ratio proportions. Statistical differences of heat trees were calculated with ‘Wilcox rank-sum test’. The ‘ggplot2 an R package’ was used for the construction of figures [25].

Network analyses were performed with NetCoMi (Network Construction and comparison for Microbiome data) between NBR versus BR samples, a comprehensive R package. Dissimilarity-based networks were generated to visualize the changes in complex microbial relationships. Edges are drawn between nodes in the case of relevant pairwise associations between bacterial taxa and edge weights referring to similarities [27].

## 3. Results

### 3.1. General Dental Information of the Participants

The study cohort comprised 12 Hungarian female and 8 Hungarian male patients. The general DMF-T of the participants in NBR group was 5.23 ± 4.5, while the general DMF-T of the BR group was 8.11 ± 4.64, which was below the values of the last Hungarian populational survey. A total of 50% of the participants were classified into age group I (mean age: 26.1 ± 1.91, mean DMF-T: 4.9 ± 4.33) and age group II (mean age: 36.3 ± 3.83, mean DMF-T: 8.9 ± 4.91). The difference of DMF-T between groups (by toothbrush change or age) was not significant with two-way ANOVA analysis (*p* > 0.05).

### 3.2. Community Diversity

Distributions of chao1 (alfa) diversity values in different patient groups (B: Day 4 of the one-week control period, F1: Day 4 of the first week, and F2: the last day of the experiment).

**Figure 2:**
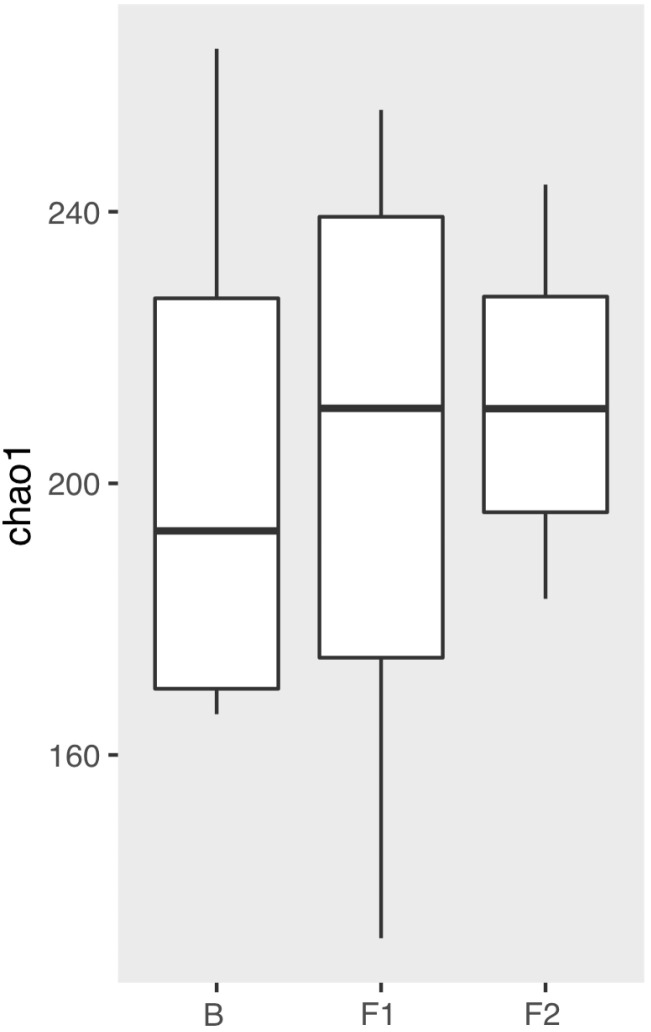
Chao1 diversity of the pooled saliva samples.

Regarding alpha diversity, Chao1 scores of the pooled saliva samples on Figure 2 showed a mild increase during F1 (follow-up 1) in comparison to the baseline (B), and to F2 (follow-up 2) group, which proved to be not significant (*p* > 0.05).

### 3.3. Correlation of DMF-T with Detected Genera

**Figure 3:**
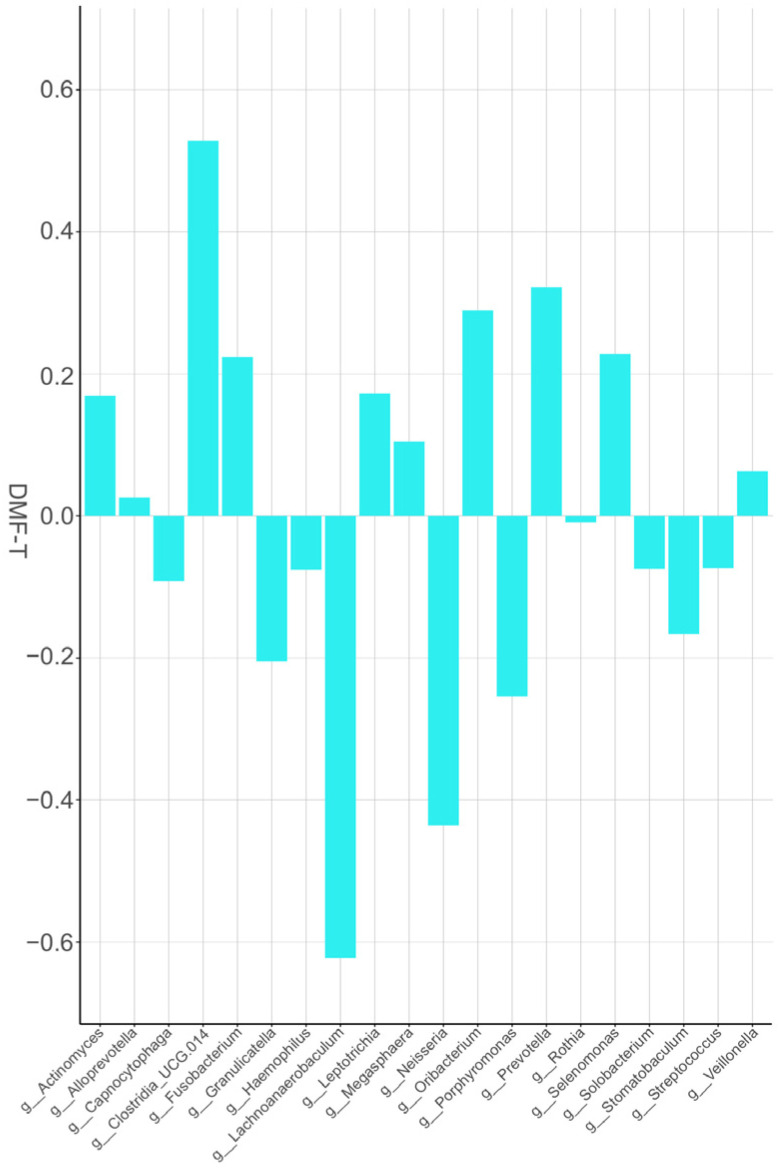
Pearson correlation analysis was performed to measure the associations between the 20 most abundant core genera of the study population and DMF-T (number of decayed, missing and filled teeth). The correlation values range from −1 to +1.

We investigated the correlations between the 20 most abundant genera [Clostridia_UCG.014 (0.528), Prevotella (0.322), Fusobacterium (0.224), Selenomonas (0.228), Oribacterium (0.289) Lachnoanaerobaculum (−0.622), Neisseria (−0.436), Porphyromonas (−0.254), and Granulicatella (−0.205)] and DMF-T values of the study population on Figure 3. 

### 3.4. The Effect of Changing the Toothbrush-Induced Remarkable Changes on the Saliva Microbiota 

**Figure 4:**
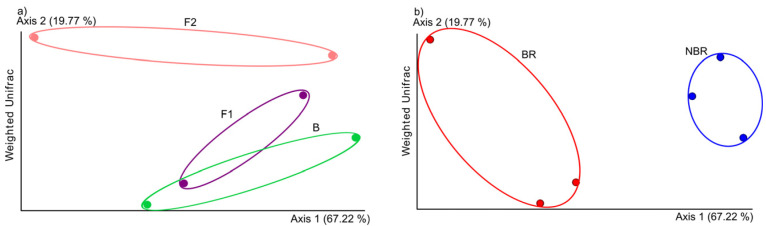

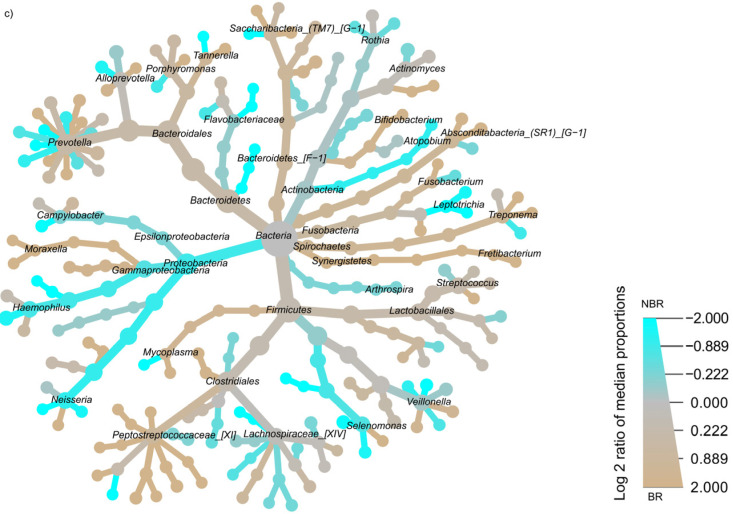
(**a**) Weighted Unifrac analysis showed overlapping of control and first week periods of chewing the gum (B green color, F1: purple color), but not in case of the second week of chewing gum usage (F2: pink color). (**b**) Weighted Unifrac analysis showed two distinct clusters separated with circles, where the red-colored dots show the BR and the blue-colored dots show the NBR group. (**c**) A heat tree showing the effects of chewing the anthocyanin gum in different groups with or without toothbrush change.

The MDS plot according to different time periods (B; F1 and F2 sample pools) showed overlapping clusters (B and F1), while the sample pools of F2 proved to be well separated from the others (Figure 4a) so the time does not seem to have remarkable effect at the beginning of treatment. 

Two distinct clusters were identified in the population represented by the sample pools, based on the toothbrush change (NBR and BR) (Figure 4b).

In order to investigate community shifts in the core-oral microbiota, a taxonomic heat tree has been made to reveal the effects of toothbrush change (Figure 4c). The Wilcoxon rank sum test showed significant differences in community compositions, but it did not show any significance of the Weighted Unifrac analysis.

### 3.5. Log_2_ Proportions of Remarkable Families and Genera of the Salivary Microbiota

**Figure 5:**
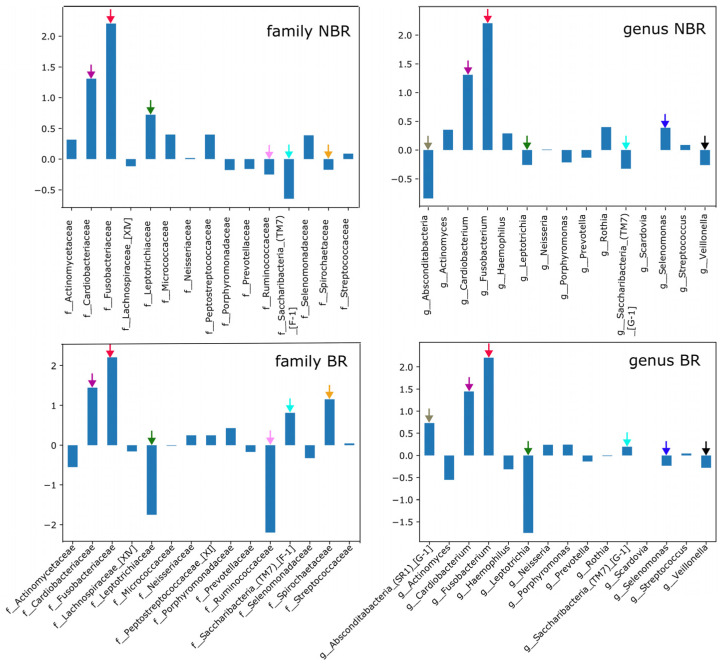
Log2 proportions of remarkable families and genera between NBR and NBR/B (upper colonnades), BR and BR/B (lower colonnades) found by Illumina sequencing in the different groups by toothbrush change after scaling. (Color codes of the most prevalent family and genera are the following: olive: genus Absconditabacteria, purple: family Cardiobacteriaceae, genus Cardiobacterium, red: family Fusobacteriaceae, genus Fusobacterium, green: family Leptotrichiaceae, genus Leptotrichia, lilac: family Ruminococcae, light blue: family and genus Saccharibacteria, yellow: family Spirochaetaceae, indigo: genus Selenomonas, black: genus Veillonella.

The changes of the log_2_ proportions of the most abundant families between the control (B) and treatment periods (BR and NBR) of both groups by toothbrush change were shown by Figure 5.

In both groups, the highest log_2_ proportions were observed for the family of Fusobacteriaceae (NBR/B: 2.207, BR/B: 2.206) and followed by Cardiobacteriaceae (NBR/B: 1.31, BR/B: 1.446), and the same tendencies could be observed in the genus Fusobacterium (NBR/B: 2.207, BR/B: 2.206) and Cardiobacterium (NBR/B: 1.31, BR/B: 1.446) during the control, as compared to the periods of chewing gum usage.

The genus Actinomyces (NBR/B: 0.354, BR: −0.553) was represented in higher levels during the NBR/B than after scaling in NBR, but in BR, opposite changes could be observed.

The log_2_ ratio of Leptotrichiaceae (NBR/B: 0.724, BR: −1.75) was less high in the control period of NBR group, and the genus Leptotrichia (NBR: −0.261, BR: −1.75) changed the same way. The opposite tendency could be observed in this family in case of BR (−1.75) and also in the NBR/B (0.724) at family level.

### 3.6. Log_2_ Proportions of Potential Biofilm Forming Genera

**Figure 6:**
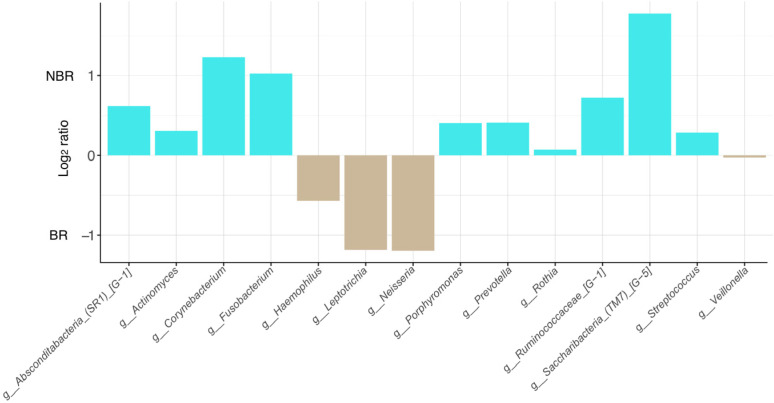
The relative frequency of biofilm-forming genera in different patient populations by toothbrush change (1–0: NBR; −1–0: BR).

The effect of toothbrush change was also examined on the relative frequency of genera taking part in the formation of biofilm (Figure 6).

The genera Absconditabacteria_(SR1)_[G_1], Corynebacterium, Fusobacterium, and Saccharibacteria (TM7) [G-5] showed the highest log_2_ ratios in the NBR, while the BR contained mainly Leptotrichia, Neisseria, and Haemophilus.

### 3.7. Relative Frequency of Detected Species

The relative frequency of the 58 identified species during the experiment (B; NBR; BR). 

The different species are presented with color codes correlating with the intensity of their relative frequencies.

We examined whether any changes can be observed in the salivary microbiota after a total plaque and calculus removal. The microbial build-up of the oral cavity started from the beginning and reflected the general oral status of the participants. 

**Figure 7:**
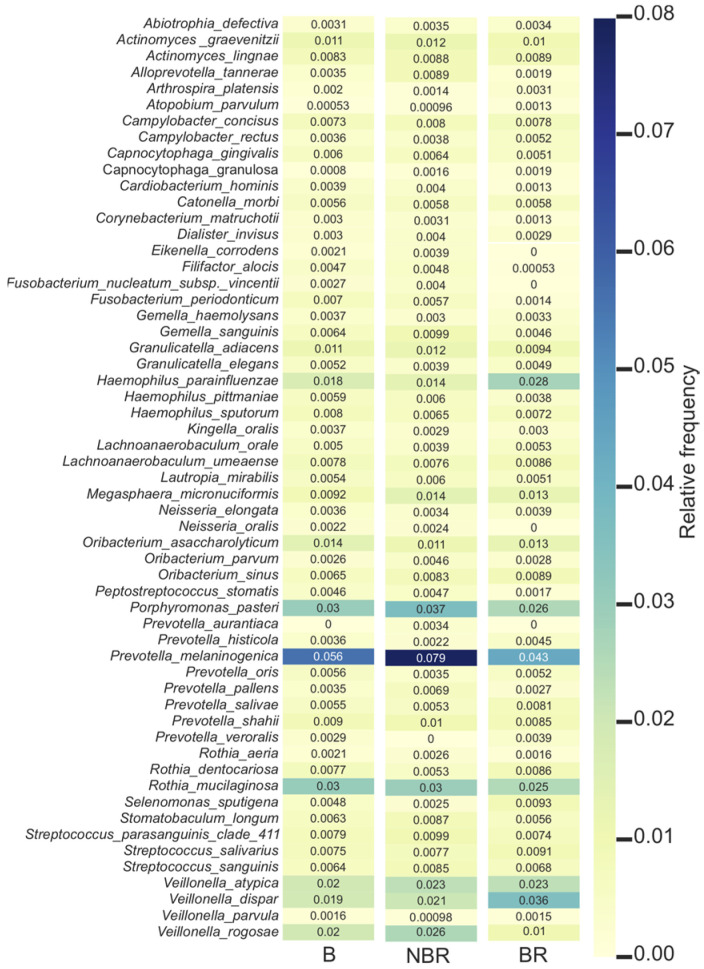
Species heatmap during the experimental periods.

The relative frequency of species in both groups and during the different treatment periods are represented on a heatmap on Figure 7. According to the heatmap, the most frequent species were the Prevotella melaninogenica, Porphyromonas pasteri, Rothia mucilaginosa, Haemophilus parainfluenzae, Veillonella atypica, Veillonella dispar, and Veillonella rogosae.

Prevotella melaninogenica had the greatest frequency in subjects without toothbrush change while chewing the gum with anthocyanin (NBR: 7.9%). Porphyromonas pasteri had a higher proportion in the case of participants who did not change their toothbrush after scaling (NBR: 3.7%) than in the other group (BR: 2.6%). Haemophilus parainfluenzae had the highest frequency in patients without brush change (BR: 2.8%), but it was less expressed in the case of the other group (NBR: 1.4%). Veillonella atypica (NBR; BR: 2.3%) and dispar (NBR: 2.1%; BR: 3.6%) were frequent in the saliva samples in both groups after the scaling. Veillonella rogosae was found to be frequent when participants did not change the toothbrush (NBR: 2.6%).

### 3.8. Microbial Networks in Groups by Toothbrush Change

Associations-based networks show the pattern of pairwise connections between the members of the 70% core taxa of BR and NBR samples by quantifying their co-occurrence using Pearson’s correlations. Edges are drawn between nodes in the case of relevant pairwise associations between bacterial taxa and edge weights referring to similarities. 

**Figure 8:**
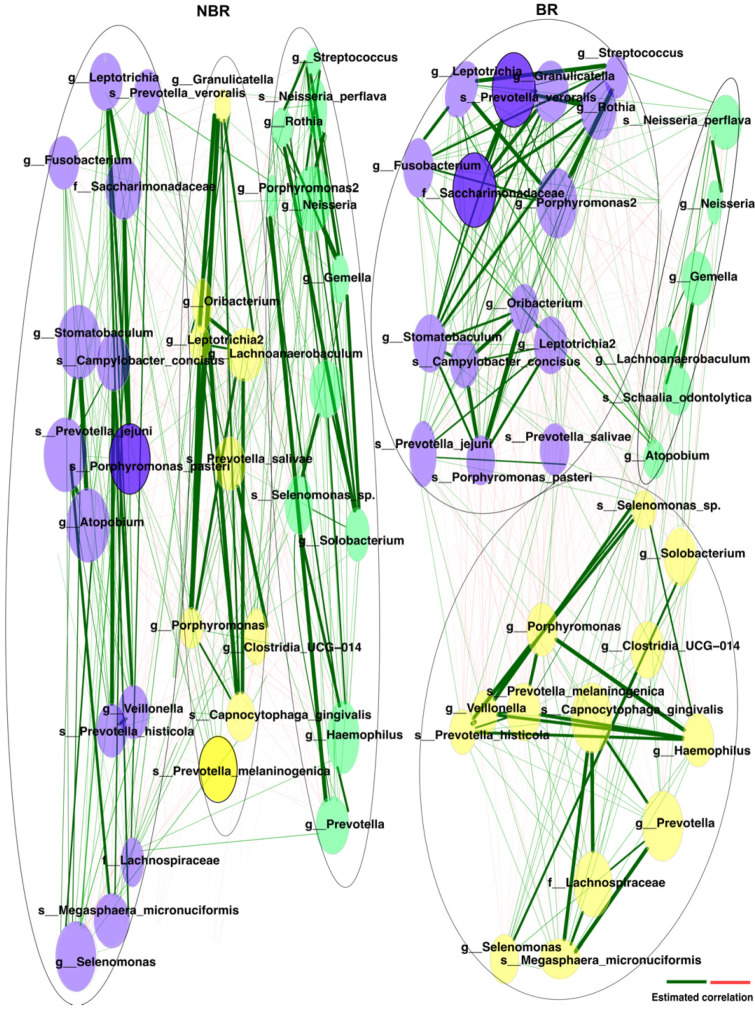
Dissimilarity-based networks were generated to compare community dynamics of the oral microbiota of BR vs. NBR samples. Green lines estimate positive correlations and red lines refer to the negative correlations. The thickness of lines shows the strength of correlation. The node colors represents different microbiota clusters: purple—cluster 1, yellow—cluster 2 and green—cluster 3.

To understand the intricate nature of the microbe–microbe and microbe–community interactions, we performed network analyses on the 70% oral-core microbiota (Figure 8). 

The total number of OTUs (operational taxonomic units) identified in the 70% core-oral microbiota was 34.

The genus Streptococcus in NBR is more likely to have a strong positive association with the genus Gemella (0.99); it also had a positive association with Neisseria (0.99), s_Neisseria perflava (0.95), and Haemophilus (0.79) in this group. The genus Streptococcus in the BR group was the part of the purple cluster, where it showed the highest positive association with Prevotella veroralis (0.95), Granulicatella (0.94), and Rothia (0.92).

## 4. Discussion

The goals of the present study were to describe the microbiota of a healthy northeast Hungarian subpopulation, as well as the effects of 2-week anthocyanin-containing chewing gum usage, supplemented by a toothbrush change in half of our study cohort.

A healthy oral cavity is characterized by a stable and diverse microbiota, while in caries the microbial diversity is reduced [8,9]. In our study, 4 days after scaling the results of Chao 1, diversity showed us that the supplementation of oral hygiene habits with anthocyanin-containing chewing gum usage can help to restore and maintain a stable and diverse microbiota, which can improve the re-building of healthier plaque [28,29,30].

In the present study, we found that the relative frequency of Clostridia_UCG.014 (0.528), Fusobacterium (0.224), and Selenomonas (0.228) in the Pearson correlation index were more frequent with higher DMF-T values (mean DMF-T: 6.9 ± 4.97). We can compare these results with the investigation of Belda-Ferre P, et al. [31], who found Clostridiales to be more abundant in caries-active patients, but did not find Selenomonas to have an etiological role in the carious process. Johansson, et al. [32] compared the microbial composition of Swedish and Romanian schoolchildren and found five Prevotella, a few Fusobacteria, and some Selenomonas species in the microbiota of Swedish caries-active adolescents, and we also found higher DMF-T values in our study. 

In our study, according to the Pearson correlation, a higher prevalence of the genus Porphyromonas can be connected to lower DMF-T values. The same tendency was observed in the study of Yasunaga, et al. [33], who compared caries-free and caries-active young adults. Caselli, et al. [34] made whole genome sequencing and detected Granulicatella, Neisseria, and Porphyromonas as health-associated bacterial genera, which are also connected to lower DMF-T values in our experiment (Granulicatella: −0.205, Neisseria: −0.436 and Porphyromonas: −0.254).

The heat tree showed significant changes with Wilcoxon rank sum test in the microbiota of groups, which were divided by the change of the toothbrush after scaling. Clustering of these groups was also supported by the Weighted Unifrac analysis. The log_2_ ratio of remarkable families and genera between groups by toothbrush change was portrayed from the most abundant family and genera of the heat tree.

The log_2_ ratio of Fusobacteriaceae and Cardiobacteriaceae at the family level were reduced in both groups by the end of experiment, and the same tendency could be observed at the genus level in Fusobacterium and Cardiobacterium. This may be beneficial as both can be related to low caries frequency [34,35,36]. Ben Lagha, et al. [37] also found that tart cherry (Prunus cerasus l.) reduced the biofilm formation ability of Fusobacterium.

There are controversial data about the prevalence of Leptotrichiaceae with caries, as they are mainly found in patients with a lower caries frequency [38], but Johansson et al. [32] found them in the Romanian caries-active population. Our beneficial results regarding the family of Leptotrichiaceae and genus Leptotrichia can be attributed mainly to the toothbrush change, as they have less log_2_ ratio in BR where the DMF-T (8.12 ± 4.64) was higher.

The observed beneficial log_2_ ratio of Actinomyces—who can take part in the carious process—can be related to both the anthocyanin in gum and toothbrush change [31].

Patients without toothbrush change were characterized by higher log_2_ ratio in Absconditabacteria, Corynebacterium, Fusobacterium, and Saccharibacteria (TM7)_[G-5] as potential biofilm-forming genera regards. The toothbrush-changing group contained genera associated with lower caries frequency, which supports further the beneficial effect of toothbrush change.

When we studied the species-level changes during our experiment, we found few Streptococcus (Streptococcus parasanguinis clade_411, Streptococcus salivarius, and Streptococcus sanguinis) in our sequencing results in a relatively low abundance. Yang, et al. [39] found the Streptococcus or Lactobacillus species to be symbionts of the oral cavity when compared caries-active and healthy people [7,8]. 

Yang et al. [39] found that different Prevotella species characterize the caries-active and caries-free people. We could not observe any statistically significant difference between the DMF-T values according to groups by changing the toothbrush after scaling, but they were characterized with different Prevotella species, which supports that they differ just in their circumstances during the experiment. Wirth et al. [5] found that the presence of Prevotella generally indicate inflammation in the oral cavity. At the species level, our findings showed that Prevotella melaninogenica was found to be more abundant in participants who did not change the toothbrush before starting the chewing gum usage.

The relative frequency of Porphyromonas pasteri was almost constant in BR group and slightly elevated in the NBR group until the end of experiment. Yasunaga et al. [33] showed that they are in a competitive interaction with the lactic acid-producing bacteria, and can lead to reduced caries formation by reducing the level of acidogenic bacteria. 

In earlier experiments, Haemophilus parainfluenzae was found in greater proportion in healthy subjects than in patients having caries [34,40]. In our study, it was found to be more frequent in the group who changed the toothbrush.

Veillonella atypica and dispar were more frequent in the saliva samples after scaling, and Veilonella dispar was found to be more abundant due to toothbrush change. Veillonella atypica and dispar can be found on healthy, carious tooth surfaces and on the tongue too, where the microbial composition is the most similar to the salivary microbiota [41]. Veillonella rogosae was found to be more frequent in patients with toothbrush change (DMF-T 8.12 ± 4.64), which was reported mainly in healthy individuals by other studies [42,43]. Veillonellae are part of the initial colonizers on enamel [44,45], and their role is suspected to be the neutralization of lactic acid, resulting in an oral niche which is less prone to caries formation [46].

The green cluster in our network analysis in NBR group represents a possible metabolic pathway of hydrogen peroxide from Streptococci. The Streptococci were in positive associations with Neisseria and Haemophilus parainfluenzae—who can use the hydrogen peroxide produced by them (e.g., Streptococcus sanguinis) [47]—hence promoting the emergence of Gram-negative bacteria [48], and perhaps creating a precarious oral niche with the Gemella genus, that could be found in caries-active patients [32]. In the BR group, the Streptococci were found in the purple cluster, where they were in stronger association with health-associated bacteria (Prevotella veroralis, Granulicatella) [49,50]. 

The limitations of our study were the relatively low sample size and the fact that the studied population had healthy oral statuses, but this was an exploratory study, and investigating the saliva of patients with higher caries prevalence is the object of further studies. The analysis of the personal, variable microbiota in saliva can be used as a predictor of certain oral diseases (caries, periodontitis) [5].

## 5. Conclusions

We can conclude that after 2 weeks of chewing gum usage, in most of the initial, early, and middle colonizers, a diverse microbiota could be detected from the saliva; however, lower levels of Prevotella melaninogenica, Porphyromonas pasteri, Fusobacterium nucleatum subsp. vincentii, and Rothia mucilaginosa could be observed in subjects who changed the toothbrush after scaling. Our results emphasize the importance of toothbrush change in reducing the level of inflammatory, anaerobic bacteria and the construction of Streptococcal network connections promoting the build-up of a healthy oral microbiota.

## Data Availability

The generated datasets supporting the reported results of the study are available from the corresponding author upon reasonable request.

## References

[1] Zhang Y., Wang X., Li H., Ni C., Du Z., Yan F. (2018). Human oral microbiota and its modulation for oral health. Biomed. Pharmacother..

[2] Nyvad B., Takahashi N. (2020). Integrated hypothesis of dental caries and periodontal diseases. J. Oral Microbiol..

[3] Le Phuong N.T., Fenyvesi F., Remenyik J., Homoki J.R., Gogolák P., Bácskay I., Fehér P., Ujhelyi Z., Vasvári G., Vecsernyés M. (2018). Protective Effect of Pure Sour Cherry Anthocyanin Extract on Cytokine-Induced Inflammatory Caco-2 Monolayers. Nutrients.

[4] Madléna M., Hermann P., Jáhn M., Fejérdy P. (2008). Caries prevalence and tooth loss in Hungarian adult population: Results of a national survey. BMC Public Health.

[5] Wirth R., Maróti G., Lipták L., Mester M., Al Ayoubi A., Pap B., Madléna M., Minárovits J., Kovács K.L. (2021). Microbiomes in supragingival biofilms and saliva of adolescents with gingivitis and gingival health. Oral Dis..

[6] Wirth R., Maróti G., Mihók R., Simon-Fiala D., Antal M., Pap B., Demcsák A., Minarovits J., Kovács K.L. (2020). A case study of salivary microbiome in smokers and non-smokers in Hungary: Analysis by shotgun metagenome sequencing. J. Oral Microbiol..

[7] Takayashu L., Suda W., Takanashi K., Iioka E., Kurokawa R., Sindo C., Hattori Y., Yamashita N., Nishijima S., Oshima K. (2017). Circadian oscillations of microbial and functional composition in the human salivary microbiome. DNA Res..

[8] Rosier T.B., Marsch P.D., Mira A. (2018). Resilience of the Oral Microbiota in Health: Mechanisms That Prevent Dysbiosis. J. Dent. Res..

[9] Paqué P.N., Herz C., Wiedemeier D.B., Mitsakakis K., Attin T., Bao K., Belibasakis G.N., Hays J.P., Jenzer J.S., Kaman W.E. (2021). Salivary Biomarkers for Dental Caries Detection and Personalized Monitoring. J. Pers. Med..

[10] He J., Tu Q., Ge Y., Qin Y., Cui B., Hu X., Wang Y., Deng Y., Wang K., Nostrand J.D.V. (2018). Taxonomic and Functional Analyses of the Supragingival Microbiome from Caries-Affected and Caries-Free Hosts. Microb. Ecol..

[11] Kumar P., Brooker M., Down S., Camerlengo T. (2011). Target Region Selection Is a Critical Determinant of Community Fingerprints Generated by 16S Pyrosequencing. PLoS ONE.

[12] Hasan N.A., Young B.A., Minard-Smith A.T., Saeed K., Li H., Heizer E.M., McMillan N.J., Isom R., Abdullah A.S., Bornman D.M. (2014). Microbial Community Profiling of Human Saliva Using Shotgun Metagenomic Sequencing. PLoS ONE.

[13] Gotelli N.J., Colwell R.K. (2011). Estimating Species Richness. Biological Diversity: Frontiers in Measurement and Assessment.

[14] Hill T.C., Walsh K.A., Harris J.A., Moffett B.F. (2003). Using ecological diversity measures with bacterial communities. FEMS Microbiol. Ecol..

[15] Fukuyama J. (2019). Emphasis on the deep or shallow parts of the tree provides a new characterization of phylogenetic distances. Genome Biol..

[16] Homoki J.R., Gyémánt G., Balogh P., Stündl L., Bíró-Molnár P., Paholcsek M., Váradi J., Fenyvesi F., Kelentey B., Nemes J. (2018). Sour Cherry Extract Inhibits Human Salivary α-amylase and Growth of Streptococcus Mutans (A Pilot Clinical Study). Food Funct..

[17] Hevesi M., Blázovics A., Kallay E., Végh A., Stéger-Máté M., Ficzek G., Tóth M. (2012). Biological Activity of Sour Cherry Fruit on the Bacterial Flora of Human Saliva in vitro. Food Technol. Biotechnol..

[18] Zinn M.K., Schages L., Bockmühl D. (2020). The Toothbrush Microbiome: Impact of User Age, Period of Use and Bristle Material on the Microbial Communities of Toothbrushes. Microorganisms.

[19] Bahador A., Lesan S., Kashi N. (2012). Effect of Xylitol on Cariogenic and Beneficial Oral Streptococci: A Randomized, Double-Blind Crossover Trial. Iran J. Microbiol..

[20] Holgerson P.L., Sjöström I., Stecksén-Blicks C., Twetman S. (2007). Dental plaque formation and salivary mutans streptococci in schoolchildren after use of xylitol-containing chewing gum. Int. J. Paediatr. Dent..

[21] Söderling E., Hirvonen A., Karjalainen S., Fontana M., Catt D., Liisa S. (2011). The effect of Xylitol on the Composition of the Oral Flora: A Pilot Study. Eur. J. Dent..

[22] Pizzo G., Licata M.E., La Carar M., Pizzo I. (2007). The effects of sugar-free chewing gums on dental plaque regrowth: A comparative study. J. Clin. Periodontol..

[23] Kejriwal S., Bhandari R., Biju T., Kumari S. (2014). Estimation of Levels of Salivary Mucin, Amylase and Total Protein in Gingivitis and Chronic Periodontitis Patients. J. Clin. Diagn. Res. JCDR.

[24] Homoki J.R., Nemes A., Fazekas E., Gyémánt G., Balogh P., Gál F., Al-Asri J., Jéréme M., Gérhard W., Babinszky L. (2016). Anthocyanin composition, antioxidant efficiency, and α-amylase inhibitor activity of different Hungarian sour cherry varieties (*Prunus cerasus* L.). Food Chem..

[25] Fidler G., Tolnai E., Stagel A., Remenyik J., Stundl L., Gal F., Biro S., Paholcsek M. (2020). Tendentious effects of automated and manual metagenomic DNA purification protocols on broiler gut microbiome taxonomic profiling. Sci. Rep..

[26] Callahan B.J., McMurdie P.J., Rosen M.J., Han A.W., Johnson A.J., Holmes S.P. (2016). DADA2: High-resolution sample inference from Illumina amplicon data. Nat. Methods.

[27] Peschel S., Müller C.L., von Mutius E., Boulesteix A.L., Depner M. (2021). NetCoMi: Network construction and comparison for microbiome data in R. Brief. Bioinform..

[28] Ko Y., Lee E.M., Park J.C., Gu M.B., Bak S., Ji S. (2020). Salivary microbiota in periodontal health and disease and their changes following nonsurgical periodontal treatment. J. Periodontal Implant. Sci..

[29] Belstrøm D., Constancias F., Liu Y., Yang L., Drautz-Moses D.I., Schuster S.C., Kohli G.S., Jakobsen T.H., Holmstrup P., Givskov M. (2017). Metagenomic and metatranscriptomic analysis of saliva reveals disease-associated microbiota in patients with periodontitis and dental caries. NPJ Biofilms Microbiomes.

[30] Simon-Soro A., Ren Z., Krom B.P., Hoogenkamp M.A., Cabello-Yeves P.J., Daniel S.G., Bittinger K., Tomas I., Koo H., Mira A. (2022). Polymicrobial Aggregates in Human Saliva Build the Oral Biofilm. mBio.

[31] Belda-Ferre P., Alcaraz L.D., Cabrera-Rubio R., Romero H., Simón-Soro A., Pignatelli M., Mira A. (2012). The oral metagenome in health and disease. ISME J..

[32] Johansson I., Witkowska E., Kaveh B., Holgerson P., Tanner A. (2016). The Microbiome in Populations with a Low and High Prevalence of Caries. J. Dent. Res..

[33] Yasunaga H., Takeshita T., Shibata Y., Furuta M., Shimazaki Y., Akifusa S., Ninomiya T., Kiyohara Y., Takahashi I., Yamashita Y. (2017). Exploration of bacterial species associated with the salivary microbiome of individuals with a low susceptibility to dental caries. Clin. Oral Investig..

[34] Caselli B., Fabbri C., D’Accolti M., Soffriti I., Bassi C., Mazzacane S., Franchi M. (2020). Defining the oral microbiome by whole-genome sequencing and resistome analysis: The complexity of the healthy picture. BMC Microbiol..

[35] Gross E.L., Leys E.J., Gasparovich S.R., Firestone N.D., Schwartzbaum J.A., Janies D.A., Asnani K., Griffen A.L. (2010). Bacterial 16S sequence analysis of severe caries in young permanent teeth. J. Clin. Microbiol..

[36] Eriksson L., Lif Holgerson P., Johansson I. (2017). Saliva and tooth biofilm bacterial microbiota in adolescents in a low caries community. Sci. Rep..

[37] Ben Lagha A., LeBel G., Grenier D. (2020). Tart cherry (*Prunus cerasus* L.) fractions inhibit biofilm formation and adherence properties of oral pathogens and enhance oral epithelial barrier function. Phytother. Res..

[38] Anderson A., Rothballer M., Altenburger M., Woelber J., Karygianni L., Lagkouvardos I., Hellwig E., Al-Ahmad A. (2018). In-vivo shift of the microbiota in oral biofilm in response to frequent sucrose consumption. Sci. Rep..

[39] Yang F., Zeng X., Ning K., Liu K.L., Lo C.C., Wang W., Chen J., Wang D., Huang R., Chang X. (2012). Saliva microbiomes distinguish caries-active from healthy human populations. ISME J..

[40] Belstrøm D., Holmstrup P., Fiehn N.E., Kirkby N., Kokaras A., Paster B.J., Bardow A. (2017). Salivary microbiota in individuals with different levels of caries experience. J. Oral Microbiol..

[41] Zhang J.S., Chu C.H., Yu O.Y. (2022). Oral Microbiome and Dental Caries Development. Dent. J..

[42] Chalmers N.I., Chen T., Hughes C.V., Kolenbrander P.E. (2011). Insights into Genus *Veillonella* in the Genomics Era. Oral Microbial Communities.

[43] Zhou P., Manoil D., Belibasakis G.N., Kotsakis G.A. (2021). Veillonellae: Beyond Bridging Species in Oral Biofilm Ecology. Front. Oral Health.

[44] Mira A. (2018). Oral microbiome studies: Potential Diagnostic and Therapeutic Implications. Adv. Dent. Res..

[45] Balachandran M., Cross K., Podar M. (2020). Single cell genomics and the oral microbiome. J. Dent. Res..

[46] Qi F., Ferretti J., Kolenbrander P.E. (2011). The Streptococcus-Veillonella Community: How Genome Sequencing Aids our Understanding of Interspecies Interaction. Oral Microbial Communities: Genomic Inquiry and Interspecies Communication.

[47] Kleinberg I. (2002). A mixed-bacteria ecological approach to understanding the role of the oral bacteria in dental caries causation: An alternative to Streptococcus mutans and the specific-plaque hypothesis. Crit. Rev. Oral Biol. Med..

[48] Ryan C., Kleinberg I. (1995). Bacteria in human mouths involved in the production and utilization of hydrogen peroxide. Arch. Oral Biol..

[49] Aas J.A., Griffen A.L., Dardis S.R., Lee A.M., Olsen I., Dewhirst F.E., Leys E.J., Paster B.J. (2008). Bacteria of dental caries in primary and permanent teeth in children and young adults. J. Clin. Microbiol..

[50] Bik E.M., Long C.D., Armitage G.C., Loomer P., Emerson J., Mongodin E.F., Nelson K.E., Gill S.R., Fraser-Liggett C.M., Relman D.A. (2010). Bacterial diversity in the oral cavity of 10 healthy individuals. ISME J..

